# Mechanism and Application of Biomaterials Targeting Reactive Oxygen Species and Macrophages in Inflammation

**DOI:** 10.3390/ijms26010245

**Published:** 2024-12-30

**Authors:** Mengxuan Yu, Shouli Wang, Doudou Lin

**Affiliations:** School of Basic Medicine, Soochow University, Dushu Lake Campus, 199 Renai Road, Suzhou Industrial Park, Suzhou 215123, China; 19891482502@163.com (M.Y.); 16665255679@163.com (D.L.)

**Keywords:** inflammation, biomaterials, reactive oxygen species, macrophages

## Abstract

Inflammation, an adaptive reaction to harmful stimuli, is a necessary immune system response and can be either acute or chronic. Since acute inflammation tends to eliminate harmful stimuli and restore equilibrium, it is generally advantageous to the organism. Chronic inflammation, however, is caused by either increased inflammatory signaling or decreased pro-anti-inflammatory signaling. According to current studies, inflammation is thought to be a major factor in a number of chronic diseases, including diabetes, cancer, arthritis, inflammatory bowel disease, and obesity. Consequently, reducing inflammation is essential for both preventing and delaying diseases. The application of biomaterials in the treatment of inflammatory illnesses has grown in recent years. A variety of biomaterials can be implanted either by themselves or in conjunction with other bioactive ingredients and therapeutic agents. The mechanisms of action and therapeutic applications of well-known anti-inflammatory biomaterials are the main topics of this article.

## 1. Introduction

Inflammation can be challenging to accurately define since it is a systemic phenomenon rather than only an acute inflammatory response caused by infection or injury [1]. The theme of the biological response to inflammation is resistance and adaptation. When resistance is more important than adaptation, the classic clinical manifestations of acute inflammation—redness, swelling, heat, pain, and dysfunction—occur. When resistance is weakened, and the stimulus persists, inflammation develops into chronic inflammation. Acute inflammation is the most common phenomenon and is triggered by a vascular response to infection or injury, which involves three main processes: increased blood flow to the site of inflammation, vasodilatation and increased permeability, and leukocytes exuding [2,3,4]. Consequently, there are two different types of natural reactions: acute and chronic inflammation.

**Figure 1:**
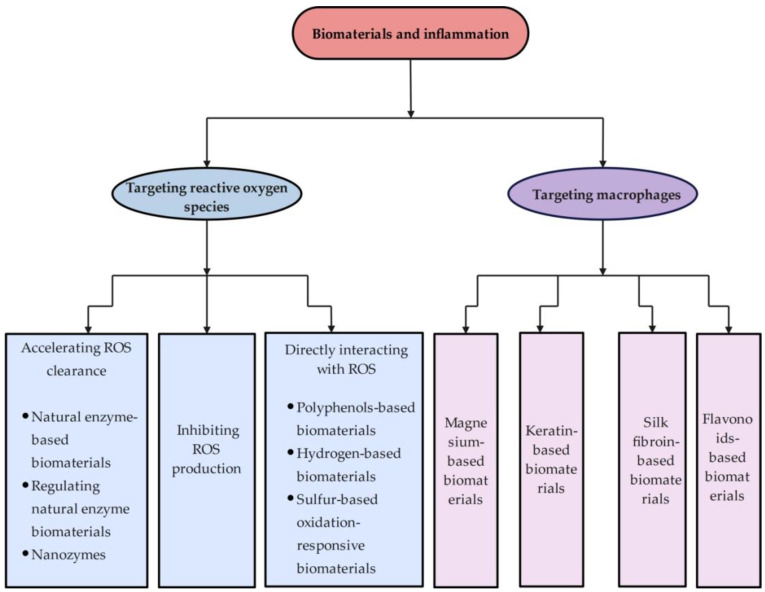
The frame diagram of this review.

Inflammation is associated with a variety of processes in cells and molecules, including aging cells, abnormal chondriosome function, decreased autophagic activity, activated inflammasomes, an unbalanced ubiquitin–proteasome system, increased responses to DNA damage, and ecological disturbances [5]. Moreover, inflammation results in an increase in reactive oxygen species (ROS), which play a significant role in the nosogenesis of chronic inflammation. Plenty of age-related illnesses have been connected to the increase in oxidative DNA damage in senile cells [6], as well as the activation of pro-inflammatory pathways by oxidative stress [7], including tumorigenesis [8], cognitive dysfunction, and neurodegenerative diseases (such as Alzheimer’s disease) [9,10]. Therefore, targeting ROS is one prospective strategy for ameliorating inflammation (Figure 1).

Macrophages, a major source of matrix metalloproteinases, chemokines, and other inflammatory mediators, drive the initial cellular response to tissue injury [11]. Indeed, the inflammatory response would be significantly reduced if macrophages are exhausted as soon as tissues are damaged [12]. However, a decrease in macrophages also delays the efficiency of tissue regeneration and healing and reduces wound debridement [13]. When the early inflammatory response diminishes, the macrophage population mostly displays wound-healing characteristics. The synthesis of several growth factors, including platelet-derived growth factor (PDGF), transforming growth factor-β1 (TGF-β1), insulin-like growth factor-1 (IGF-1), and vascular endothelial growth factor-α (VEGF-α) that promote blood vessel development and cellular proliferation, characterizes these phenotypes [14,15,16,17,18]. Monocytes and/or macrophages then take over, primarily for their anti-inflammatory properties [19]. Macrophages are also essential innate immune cells assisting tissues recover from acute inflammation by phagocytizing foreign substances, modifying extracellular matrix (ECM), promoting angiogenesis, and repairing and restoring tissue homeostasis. Research on the connection between macrophages and inflammation is ongoing. Targeting macrophages in the relief of inflammation appears promising due to their vital roles (Figure 1).

Biomaterials are substances that can be employed to direct any therapeutic or diagnostic procedure in human or veterinary medicine, either independently or in conjunction with other substances, by controlling how they interact with the components of a biological system [20]. Biomaterials have a tendency to induce an inflammatory response by causing a foreign body reaction (FBR) around the implant when they are embedded into the body [21]. Therefore, in order to prevent FBRs and regulate immune responses, biomaterials should promote the synthesis of anti-inflammatory cytokines [interleukin (IL)-10], hinder the generation of oxidative stress species (ROS and nitrites), and regulate the synthesis of pro-inflammatory cytokines [ tumor necrosis factor-α (TNF-α), IL-1 and IL-6]. In the meantime, contemporary research has shown that inflammation occurs in a wide range of illnesses, such as cancer, myocardial ischemia [22,23], acute cerebral stroke [24], arterial hypertension [25], and osteoarthritis [26]. Consequently, inflammation has gained popularity as a topic in disease studies. Since different biomaterials have been demonstrated to have differing effects on inflammatory illnesses, research and application of anti-inflammatory biomaterials have received a lot of interest recently [27,28,29].

## 2. Biomaterials Targeting Scavenging Reactive Oxygen Species

### 2.1. ROS, Inflammation and Biomaterials

Chemicals generated by inadequate reduction of molecular oxygen are the primary source of ROS, which are crucial mediators of inflammation [30]. ROS primarily consist of hydrogen peroxide (H_2_O_2_), monoclinic oxygen (^1^O_2_), superoxide anion (O_2_^−^), and hydroxyl radicals (·OH) [31].

In a physiological sense, the removal and yield of ROS are in dynamic balance to maintain a lower ROS level [32]. Both excessive ROS production and insufficient ROS elimination contribute to inflammation. Additionally, studies have shown that ROS affects a number of inflammatory signaling pathways, including the nod-like receptor family pyrin domain-containing 3(NLRP3), nuclear factor kappa B(NF-κB), the mitogen-activated protein kinase (MAPK), Janus kinase/signal transducer and activator of transcription (JAK/STAT), nuclear factor erythroid 2-related factor 2 (Nrf2), and phosphatidylinositol 3-kinase PI3K/protein kinase B (PKB/AKT) [33].

Due to the tight relationship between ROS and inflammation, it is now reasonably feasible to treat inflammation with biomaterials that scavenge ROS [34]. By modifying the generation and elimination of ROS both directly and indirectly, biomaterials scavenging ROS can exert an anti-inflammatory effect. Meanwhile, the adverse effects of conventional anti-inflammatory drugs, such as renal failure and gastrointestinal and cardiovascular complications caused by corticosteroids and non-steroidal anti-inflammatory drugs [35], have also led to the development of biomaterials that scavenge ROS as a supplementary measure for inflammation therapy [36]. Hence, developing and manufacturing biomaterials that scavenge ROS is a crucial step in restoring ROS balance, managing inflammatory responses, and protecting the host.

Based on different mechanisms of action, biomaterials with ROS-scavenging properties have been developed in recent years and can be classified into three categories: enzymatic biomaterials that catalyze and speed up ROS scavenging, biomaterials that interact directly with ROS, and biomaterials that block the source of ROS to reduce ROS output.

### 2.2. Classification of Biomaterials Targeting Scavenging ROS

#### 2.2.1. Accelerating ROS Clearance

Natural enzyme-based biomaterials, regulating natural enzyme biomaterials, and nanozymes are among the biomaterials used to catalytically speed up the removal of ROS (Table 1).

##### Natural Enzyme-Based Biomaterials

The research has proven the imbalance of the ROS scavenging system can lead to a variety of inflammatory disorders that can be treated with natural enzymes [36], including glutathione peroxidase (GPX), catalase (CAT), and superoxide dismutase (SOD) [37,38,39,40].

However, inherent properties of natural enzymes, like their short half-lives and poor stability under operating circumstances, typically limit their therapeutic efficacy [41]. To capitalize on natural enzymes, scientists have therefore developed and manufactured a number of biomaterials based on them. With the antioxidant qualities of CAT and SOD, Zhang et al. synthesized a nanorod [CeO_2_ (CeO_2_@PP)]. The investigations revealed that the nanorods assisted in polarizing macrophages from M1 to M2 and lowered ROS to mitigate inflammation [42]. Meanwhile, a type of nanoparticle with CAT-like and SOD-like activity was designed named CeO₂@PAA@RGD. By stimulating macrophages to switch from M1 to M2 and removing ROS, the nanoparticles could ameliorate inflammation [43].

##### Regulating Natural Enzyme Biomaterials

Regulating natural enzyme biomaterials reinforced physiological enzymatic ROS defensive mechanism to obliterate ROS, as well as by enhancing the physiological activity of natural scavenging ROS enzymes to alleviate inflammation. For instance, Chen et al. constructed a material named Se-MBG through the mixture of mesoporous bioactive glass with selenium. The results suggested it could regulate macrophage polarization and scavenge excessive ROS of cells to promote bone regeneration and diminish inflammation, particularly via enhancing the expression of GPX-4 in the macrophages [44].

##### Nanozymes

Nanozymes are nanoparticles with the ability to replicate the catalytic activity of particular biological enzymes. Excellent stability, versatility, and other properties are possessed by these nanozymes [45]. ROS nanozymes, containing Ce, Mn, Cu, and other elements have been exploited and fabricated in studies to catalytically remove ROS [36]. As an illustration, Duan et al. set up a nanoparticle made of mesoporous bioactive glass and cerium (Ce-MBGN), which could remove ROS of dental pulp cells and macrophages while preventing macrophage 1s from transforming to produce anti-inflammatory effects [46]. Additionally, the other investigation combined the nanozymes MnO₂ with gelatin to fabricate the substance (MnO₂@PDA-BGs/Gel). Which could eliminate intracellular ROS and manage the reversal of inflammation to hasten wound healing [47].

With their relative affordability, enhanced stability, and other benefits, nanozymes combined the properties of nanomaterials with the activity of natural enzymes, demonstrating remarkable potential. Notwithstanding its many benefits, the poor selectivity of substrate is a problem that needs to be fixed. 

**Table 1:** The classification and action of biomaterials accelerating ROS clearance.

Classification	Actions in Diseases	References
Natural enzyme-based biomaterials	(1) Promoting polarization of macrophages from M1 to M2 (2) Reducing ROS (3) Increasing expression of anti-inflammatory cytokines such as CD206, VEGF, and IL-10	[41,42,43,48]
Regulating natural enzyme biomaterials	(1) Scavenging excessive ROS of cells (2) Controlling macrophage polarization	[44,49]
Nanozymes	(1) Lessening ROS of cells and macrophages (2) Preventing macrophage 1s from transforming	[46,47]

#### 2.2.2. Directly Interacting with ROS

Polyphenol-based biomaterials, hydrogen-based biomaterials, sulfur-based oxidation-responsive materials, and other biomaterials are used to directly interact with ROS (Table 2).

##### Polyphenol-Based Biomaterials

Polyphenols are naturally occurring plant compounds with potent antioxidant, anti-tumor, and anti-inflammatory qualities [50]. Research indicates that its anti-inflammatory properties are closely linked to its antioxidant function [50].

One such substance is resveratrol (Res). Hindering the production of ROS and NO to exert anti-inflammatory and antioxidant abilities, Res simultaneously increases the amount of anti-inflammatory chemicals so as to minimize inflammation [51]. Tang et al. produced resveratrol nanoparticles to speed up infectious wound healing by incorporating res into nanoparticles via the Mannich reaction and functionalizing them with phenylboric acid. Studies proved that Res NPs had an anti-inflammatory effect by limiting the expression of nitric oxide (NO), IL-6, and TNF-α [52]. Considering these benefits of creating a resveratrol nanoparticle, we can conclude that resveratrol-based materials have countless potential in tissue engineering. In addition, curcumin is a polyphenol. Mahdieh Alipour et al. constructed and evaluated a curcumin-based compound that demonstrated potential for alleviating infectious and inflammatory responses while aiding the mineralization of the dentin-pulp complex [53]. When blending curcumin with supramolecular nanoparticles, a particle system was developed that assisted in polarizing macrophages and reducing inflammation and oxidative stress [54]. The polyphenols also include epigallocatechin gallate (EGCG). The nano-platforms designed by researchers Ming et al. have various functions, such as removing ROS, modifying the immune system, and stimulating the formation of blood vessels [55]. Furthermore, a novel scaffold was manufactured using EGCG and hydrogen peroxide dual-responsive borosilicate glass (BSG), which contributed to controlling the inflammatory response of diabetic bone defects [56].

##### Hydrogen-Based Biomaterials

Hydrogen (H_2_) can directionally suppress ·OH and the peroxynitrite anion (·ONOO^−^) while guaranteeing the involvement of other significant ROS and reactive nitrogen species (RNS) in other regular cellular signaling pathways; consequently, it serves as a superior antioxidant for treating oxidative stress in comparison to other antioxidants [57]. In the meantime, in accordance with the observational study, its anti-oxidation mechanism may be mostly related to genetic expression rather than directly scavenging radicals. As an instance, transcription factor Nrf2, which is regulated by H_2_, has been shown in various kinds of studies to alleviate NF-κB-induced inflammation [58,59,60]. Another study revealed that H_2_ can alter the MAPK and Toll-like receptor 4 (TLR4) signaling pathways [61]. Furthermore, H_2_ can hinder the invasion of neutrophils, M1 macrophages, and lymphocytes during inflammation, which in turn diminishes pro-inflammatory cytokines such as IL-1β, IL-6, IL-8, and TNF-α [62,63,64], high mobility group box 1(HMGB1) [65], and interferon-γ(IFN-γ) [66], as well as chemokines macrophage inflammatory protein 1 and 2 (MIP1 and MIP2). In conclusion, these actions evidenced the anti-inflammatory and anti-oxidant qualities of H_2_. As a consequence, the growing popularity of hydrogen-based biomaterials for the treatment of diseases is attributed to the anti-oxidative and anti-inflammatory qualities of H_2_.

Then, photo-driven H_2_-evolving liposomal nanoplatform (Lip NP) was produced, containing the upconversion nanoparticle (UCNP) that was coupled with gold nanoparticles (AuNPs) via a ROS-responsive linker, following encapsulating them in the liposomal system in which chlorophyll a (Chl a) was embedded in the lipid bilayer. According to the study, it effectively inhibited the expression of pro-inflammatory factors, including IL-1 and IL-6, and reduced the excessive generation of ROS in macrophages [67]. Alao and Wan et al. designed a multicomponent nanoreactor (NR) encapsulating gold nanoparticles, l-ascorbic acid, and chlorophyll a into a liposomal (Lip) system that could produce H_2_ gas to lessen inflammation when absorbing photons. The findings showed the overproduction of ROS and pro-inflammatory cytokines, including IL-6 and IL-1, in the lipopolysaccharide (LPS)-stimulated macrophages could be dropped in the Lip NR groups [68].

##### Sulfur-Based Oxidation-Responsive Biomaterials

Sulfur is necessary for the regulation of many redox processes and signaling pathways [69,70,71]. Scavenging ROS is one of its important functions, which heavily depends on the thioredoxin (Trx) system [72,73]. As a result, there has been a significant increase in interest in the potential of sulfur compounds to treat and prevent inflammatory diseases due to their powerful ability to scavenge ROS. Sulfur compounds contain hydrogen sulfide, thioethers/sulfoxides, thioacetals/thioketals, and other chemicals.

Thioethers are among the substances containing sulfur. Polythioether/polysulfide can directly function via lowering inflammation since thioethers are readily transformed into sulfoxides or sulfones when exposed to raised concentrations of ROS [74]. Certain sulfoxides will continue to be oxidized to sulfones by ROS [75,76], such as dimethyl sulfoxide (DMSO), which can reduce inflammation by decreasing macrophage polarization, cytokine production, and lymphocyte activation and proliferation [77]. When thioethers are oxidized in an aqueous environment, their solubility significantly increases as a result of their hydrophobic structures changing into the hydrophilic structures of sulfoxide and sulfone. Accordingly, polythioether/polysulfide may be used as a carrier to address ROS and incorporate anti-inflammatory drugs to enhance the interaction between the drug delivery system and ROS scavenging. By means of attaching electrospun thioether to hyaluronic acid (FHHA-S/Fe) to form a nanofibrous hydrogel. As demonstrated by the results, the hydrogel reduced the synthesis of pro-inflammatory chemokines like IL-1β, IL-6, and TNF-α and enhanced their conversion into anti-inflammatory chemokines like IL-4 and IL-10, which might rapidly eliminate ROS in the early stages of inflammation and reduce swelling [78]. Additionally, Yoo et al. combined thiol-Ene with thiol-Michael to create core-cross-linked nanoparticles (NPs) from polysorbate 80 (PS80). The study suggested that NPs could inhibit inflammatory responses in nerves and the secondary spread of injury based on magnetic resonance imaging and histology in the cortical impact mouse model of traumatic brain injury (TBI) [79].

Hydrogen sulfide (H₂S) is a corrosive, water-soluble, flammable, colorless, and poisonous gas [80]. In addition to directly scavenging ·ONOO^−^ and NO [81], H_2_S scavenging ROS also indirectly improves the defense mechanism of anti-oxidation by altering the Nrf2 signaling pathway [82], the SR-A signaling pathway [80] (SR-A is a component of the scavenger receptor of A macrophage), and GSH levels [83,84,85]. Thus, these sulfur compounds have an infinite ability to alleviate aberrant ROS-induced inflammation. Employing peroxy-thiocarbamate (PTCM, an H_2_S donor) encapsulated in a ROS-responsive polymer [methoxy poly (ethyleneglycol)-poly(L-methionine) (mPEG-PMet)] and loaded into a temperature-sensitive hydrogel [methoxy poly (ethyleneglycol)-polyalanine-polyphenylalanine (mPEG-PA-PP)], Dong et al. developed an H_2_S delivery system that has been shown to help prevent inflammation and oxidative stress while protecting nerve cells and angiogenesis in peripheral nerve injury (PNI) [86]. Meanwhile, a hydrogel dressing via encapsulating PTCM in PMet to form a nanoparticle, which was subsequently combined with F127-P(Asp-NHS), a thermosensitive injectable hydrogel composed of N-hydroxy succinimide and L-aspartic acid, to create the dressing. According to the study, it could reduce oxidative stress and infection while reducing inflammation to aid in wound healing [87]. Additionally, a poly (γ-glutamic acid) (PGA) hydrogel encapsulated with ciprofloxacin (Cip), anthocyanins (Ant), and a keratin-based H_2_S donor (KTC) was designed. This hydrogel was able to release H_2_S, thereby promoting the healing of diabetic wounds by lessening inflammation, contributing to angiogenesis and collagen deposition, and enhancing anti-inflammatory and antioxidant properties [88].

**Table 2:** The classification and action of biomaterials directly interacting with ROS.

Classification		Actions in Diseases	References
Polyphenol-based biomaterials	Resveratrol-based materials	(1) Inhibiting the expression of NO, IL-6, and TNF-α to play an anti-inflammatory role (2) Scavenging ROS	[52]
	Curcumin-based material	(1) Reducing infectious and inflammatory responses (2) Alleviating inflammatory response and oxidative stress (3) Promoting the polarization of macrophages	[53,54]
	Epigallocatechin gallate (EGCG)	(1) Possessing the ability to scavenge ROS (2) Regulating inflammatory response and immunomodulating the human body (3) Promoting angiogenesis	[55,56]
Hydrogen-based biomaterials	-	(1) Decreasing the overproduction of ROS in macrophages (2) Inhibiting the expression of pro-inflammatory factors such as IL-1β and IL-6	[67,68]
Sulfur-based oxidation-responsive materials	Thioether/sulfoxide- contained biomaterials	(1) Scavenging ROS (2) Mitigating inflammation via inhibiting the production of pro-inflammatory chemokines, including IL-1β, IL-6, and TNF-α (3) Enhancing the conversion of anti-inflammatory chemokines, including IL-4 and IL-10	[78,79]
	Hydrogen sulfide (H₂S)	(1) Inhibiting inflammation and oxidative stress (2) Promoting angiogenesis (3) Remodeling inflammation, strengthening anti-inflammatory and anti-oxidant effects	[86,87,88]

#### 2.2.3. Inhibiting ROS Production

To mitigate ROS creation, nicotinamide adenine dinucleotide phosphate oxidases (NOX), xanthine oxidoreductase (XO), lipoxygenases (LOX), or NOX-involved ROS yield are generally blocked. NADPH oxidases modify the cellular redox characteristic by modulating the ROS content in cells [89]. It has already been established that one of the main mechanisms connecting NOX4 and ferroptosis is the modification of ROS production to influence the iron metabolic pathway. Consequently, targeted NOX that affects macrophages in inflammation tends to influence ferroptosis, oxidative pressure, and inflammation. Thus, Zhen et al. developed a microsphere capsule (mPEG-TK-GLX@PVA-MMA) that responded to ROS and reduced inflammation while obstructing ferroptosis and ROS generation [90].

In conclusion, the intention of biomaterials that prevent the production of ROS is to fundamentally block its generation. Nevertheless, being short of tissue specificity and having insufficient bioavailable ability in some organs, which can reduce the effectiveness of treatment, are among the more crucial issues with this novel biomaterial. Furthermore, it is still being investigated how to restrict ROS production fails to inhibit ROS normally functioning.

### 2.3. Manufacture, Application and Action

For periodontitis, the researchers covered nanoceria with chlorin e6 (Ce6), a red light-excited photosensitizer, to create a multifunctional nanocomposite for periodontitis. Through the use of nanoceria, this nanocomposite scavenges remaining ROS, suppresses M1 macrophage polarization, and stimulates M2 macrophage polarization, all of which control the immune system [91]. Additionally, a study created microspheres (Se-nHA/PC) by electrostatically injecting proanthocyanidins (PC) and sericin-hydroxyapatite nanoparticles (Se-nHA NPs) into sericin/sodium alginate (Se/SA). These microspheres demonstrated an anti-inflammatory and antioxidant effect by stimulating M2 macrophage polarization, suppressing M1 macrophage polarization, scavenging ROS, and preventing the synthesis of pro-inflammatory chemicals [92]. On top of that, Shi et al. established a resveratrol-loaded liposomal system (Lipo-RSV) that attenuated pro-inflammatory cytokines such as TNF-α, IL-1β, and IL-6. Likewise, it activated p-STAT3 and deactivated p-STAT1 to convert M1 macrophages to M2 and eliminate ROS. They also blocked inflammasomes and the NF-κB signal for an anti-inflammatory action [93].The above research suggests that using biomaterials scavenging ROS to treat periodontitis is both possible and promising (Table 3).

For diabetic wound healing, Tu et al. combined water-soluble PPGA polymers with manganese dioxide (MnO₂) nanozymes that had been modified with hyperbranched poly-L-lysine (HBPL) to create a hydrogel. This hydrogel is then combined with pravastatin sodium, a chemical that contributes to the production of NO, to create HMP hydrogel. By reducing the expression of inflammatory cytokines like IL-1, IL-6, TNF-α, and CXC ligand 1 (CXCL-1), the HMP hydrogel can aid in the reduction of inflammation. It can also decrease ROS and neutrophil infiltration, increase IL-4 and IL-10, and aid in the polarization of M2 macrophages [94]. Another study reveals the design of nanosheets (PtCuTe) by using Pt(acac)₂, Cu (CH₃COO)₂, and H₆TeO₆ as starting metals, citric acid as a reducing agent, and Mo (CO)₆. It primarily reduces inflammation by increasing M2 macrophages, decreasing M1 macrophages, and inhibiting IL-1, IL-6, and TNF-α production [95]. Furthermore, research findings demonstrate that to manufacture cobalt ion-doped bioactive nanozymes (CoNZs) using a modified sol–gel technique under alkaline circumstances utilizing poly(ethyleneglycol) as a structural template, which down-regulated pro-inflammatory molecules and promptly scavenged ROS [96]. Qi and colleagues created a thermoreversible hydrogel that encapsulated cerium (Ce)-doped Linde type A (LTA) zeolite-based nanoparticles (Ce@LTA-NPs) by loading it with Pluronic F127 and chitosan. Its unique three-dimensional crystal structure and regular pore shape provide an anti-inflammatory action by absorbing explosive inflammatory cytokines in acute inflammatory situations. As well, by replicating the actions of CAT and SOD in cells, it lessened excessive ROS generation [97]. To generate the innovative system (GelMA@Mg-POM), a novel bioactive nanozyme (Mg-POM) made of magnesium ions doped with molybdenum was added to a gel of methacrylate and gelatin. Which can scavenge ROS, ameliorate the inflammatory environment, induce the shift of macrophage phenotype to M2, and diminish macrophage-associated inflammatory responses [98]. Simultaneously, using a modified hydrothermal process, Chen et al. produced a 2D copper antioxidant nanozyme (Cu NS). In addition to reducing inflammation by inhibiting the synthesis of NO, it can also inhibit the expression of inflammatory cytokines such as TNF-α, IL-6, and inducible nitric oxide synthase (*iNOS*). Furthermore, it was clear that macrophages increased anti-inflammatory molecules such as arginase (*Arg*)-1, *CD163*, and *CD206*. Not only did these factors assist in polarizing M2 macrophages, but they also weakened the polarization of M1 macrophages [99]. Biomaterials scavenging ROS appear to be a promising therapeutic method for the future treatment of diabetic wound healing, according to these results and other ongoing research (Table 3).

For osteoarthritis, OHA/HAADH@SeNPs hydrogels are a novel injectable hydrogel developed by Hu et al. that transports selenium nanoparticles by crosslinking oxidized hyaluronic acid (OHA) with hyaluronic acid-adipic acid dihydrazide (HA-ADH). Redox equilibrium was restored, and IL-1β and TNF-α levels were reduced by targeting GPX-1 and removing ROS. These actions also reduced inflammatory responses [100]. Additionally, the study conducted by Kumar produced PEG-MnO₂, a ROS-scavenging nanoparticle, by adding polyethylene glycol (PEG) to MnO₂ nanoparticles (NPs) to improve their stability. MnO₂ NPs were generated through a redox reaction between potassium permanganate (KMnO₄) and poly (allyl amine) hydrochloride (PAH). It did not cause inflammatory cascades and instead reduced TNF-αexpression in classically stimulated macrophages [101]. Another study discovered that layered double hydroxides (LDHs) and TAGel, which were produced by crosslinking gelatin with tannic acid (TA), could be encased to create a bioactive material called LDH@TAGel. Through the Nrf2/Keap1 system and the PI3K-Akt pathway, LDH@TAGel can shield chondrocytes from the oxidative stress brought on by inflammatory reactions and apoptosis [102]. An artificial nanozyme called hydrogel-loaded iron-doped zeolitic imidazolate framework-8 (Fe/ZIF-8/Gel) centrase was developed to scavenge endogenous excess ROS. This nanozyme may reduce inflammatory responses by inhibiting the expression of MMP13 and IL-6. Additionally, it may lessen inflammation by maintaining intrinsic SOD activity and inhibiting the production of intracellular malondialdehyde (MDA) [103]. Wei et al. synthesized Cu-EGCG nanosheets, a metal-polyphenol nanoformulation, by combining EGCG with copper ions. These nanosheets contain the anti-inflammatory and antioxidant qualities of Cu2+. Apart from aiding macrophages in transitioning from M1 to M2 polarization, it may also reduce the levels of inflammatory cytokines such as TNF-α, *iNOS*, IL-1β, and IL-6. Controlling the activation of Nrf2 and the inactivation of NF-κB may also enhance its anti-inflammatory properties [104]. According to these findings, osteoarthritis may be treated using biomaterials that eliminate ROS (Table 3).

For inflammatory bowel disease (IBD), researchers Zhang and colleagues assembled a mimic SOD/CAT nanomedicine containing a hydrogen peroxide-eliminating nanomatrix and a free radical scavenger, Tempol (Tpl). After that, an oxidation-responsive b-cyclodextrin material named OxbCD is produced, and OxbCD nanoparticle loading with Tpl (Tpl/OxbCD NP) is manufactured. They found that inflammation can be lessened by restricting the synthesis of inflammatory cytokines such as TNF-α, IFN-γ, and IL-1β [105]. A novel CAT mimetic based on manganese metal-organic frameworks (Mn-MOFs) embedded in microfluidic microcapsules was engineered concurrently by another study. The findings illustrated a considerable improvement in SOD activity and a decline in the expression of inflammatory cytokines, including TNF-α, IL-1α, IL-1β, and IL-6, which triggered the anti-inflammatory effect [106]. It is encouraging that this biomaterial can be used to treat inflammatory bowel disease, even if this review only covers a small portion of the many investigations on biomaterials scavenging ROS for this purpose (Table 3).

In conclusion, research on the anti-inflammatory properties of ROS-scavenging biomaterial still requires improvement, as well as a more thorough and sufficient synopsis of the ROS and inflammation is still required [107]. Such studies will contribute to the design and sufficient application of biomaterials scavenging ROS. Details on the application of scavenging ROS biomaterials are given in Table 3.

**Table 3:** Application of biomaterials scavenging ROS in diseases.

Disease	Designed Materials	Actions	References
Periodontitis	CeO_2_@Ce6	(1) Removing remaining ROS (2) Suppressing M1 macrophage polarization, and promoting M2 macrophage polarization	[91]
	Se-nHA/PC	(1) Stimulating M2 macrophage polarization, suppressing M1 macrophage polarization (2) Scavenging ROS (3) Preventing the synthesis of pro-inflammatory chemicals	[92]
	Lipo-RSV	(1) Attenuating the production of pro-inflammatory cytokines such as TNF-α, IL-1β, and IL-6 (2) Activating p-STAT3 and deactivating p-STAT1 to convert M1 macrophages to M2 and eliminate ROS (3) Blocking inflammasomes and the NF-κB signal for an anti-inflammatory action	[93]
Diabetic wound healing	HMP hydrogel	(1) Suppressing the expression of inflammatory cytokines like IL-1, IL-6, TNF-α, and CXCL-1 (2) Decreasing ROS and neutrophil infiltration (3) Contributing to the production of IL-4 and IL-10 (4) Aiding the polarization of M2 macrophages	[94]
	PtCuTe nanosheets	(1) Increasing the number of M2 macrophages, decreasing the number of M1 macrophages (2) Inhibiting IL-1, IL-6, and TNF-α production (3) Scavenging ROS	[95]
	CoNZs	(1) Down-regulating pro-inflammatory molecules (2) Removing ROS	[96]
	A thermoreversible hydrogel	(1) Absorbing explosive inflammatory cytokines in acute inflammatory situations to provide an anti-inflammatory environment (2) Replicating the actions of CAT and SOD in cells to lessen excessive ROS generation	[97]
	GelMA@Mg-POM	(1) Scavenging ROS (2) Ameliorating the inflammatory environment (3) Promoting the shift of macrophage phenotype to M2 (4) Diminishing macrophage-associated inflammatory responses	[98]
	Copper enzyme	(1) Inhibiting the synthesis of NO to reduce inflammation (2) Restricting the expression of inflammatory cytokines such as TNF-α, IL-6, and *iNOS* (3) Increasing the expression of anti-inflammatory molecules of macrophages such as arginase (*Arg*)-1, *CD163*, and *CD206* (4) Promoting the polarization of M2 macrophages, weakening the polarization of M1 macrophages	[99]
Osteoarthritis	OHA/HAADH@SeNPs hydrogels	(1) Restoring redox equilibrium (2) Decreasing IL-1β and TNF-α levels by targeting GPX-1 and removing ROS (3) Reducing inflammatory responses	[100]
	PEG-MnO₂	(1) Failing to cause inflammatory cascades (2) Reducing TNF-α expression in classically stimulated macrophages	[101]
	LDH@TAGel	(1) Protecting chondrocytes from the stress caused by inflammatory reactions and apoptosis through the Nrf2/Keap1 system and the PI3K-Akt pathway (2) Reducing the cartilage deterioration and degeneration brought on by anterior cruciate ligament transection (ACLT)	[102]
	Fe/ZIF-8/Gel centrase	(1) Reducing inflammatory responses by inhibiting the expression of MMP13 and IL-6 (2) Lessening inflammation by maintaining intrinsic SOD activity and inhibiting the production of intracellular MDA	[103]
	Cu-EGCG nanosheets	(1) Contributing to macrophages in transitioning from M1 to M2 polarization, (2) Reducing the levels of inflammatory cytokines such as TNF-α, *iNOS*, IL-1β, and IL-6. (3) Controlling the activation of Nrf2 and the inactivation of NF-κB	[104]
IBD	SOD/CAT nanomedicine	(1) Restricting the synthesis of inflammatory cytokines such as TNF-α, IFN-γ, and IL-1β	[105]
	CAT mimetic	(1) Decreasing the expression of inflammatory cytokines, including TNF-α, IL-1α, IL-1β, and IL-6 (2) Improving SOD activity	[106]

## 3. Biomaterials Targeting Macrophages

### 3.1. Macrophages, Inflammation and Biomaterials

Macrophages are divided into two subgroups, M1 and M2, according to their plastic phenotype. Each of the four subpopulations that make up the M2 macrophage subtype—M2a, M2b, M2c, and M2d—has a unique activatory mechanism and purpose. In the meantime, M2 is alternatively activated and primarily promotes tissue remodeling and repair [108,109,110] to defend and preserve tissue homeostasis [111,112], while M1 is classically activated and mostly pro-inflammatory, dominating inflammation. Pro-inflammatory cytokines, including IL-1β, IL-6, IL-12, TNF-α, and IFN-γ, are produced by M1 macrophages [113]. Conversely, M2 macrophages generate anti-inflammatory cytokines like TGF-β, IL-10, and IL-13 [114]. IL-1β is the strongest pro-inflammatory cytokine. It contributes to many autoimmune diseases, inflammatory responses, and the persistence of pain in chronic disorders [115]. The cytokine family IL-10 is quite distinct from IL-1. They can trigger the host to conduct several defensive actions, including preventing bacterial and viral infections and preserving the integrity of epithelial tissue. IL-10 notably benefits wound healing and shields the host from the harmful effects of infection or inflammation [116]. Cytokines, including IL-6 and the TGF-β superfamily, also directly control angiogenesis [117,118].

When the body is exposed to biomaterials, the immune system may cause an inflammatory response. Acute inflammation brought on by neutrophils first appears in the human body to promote more immune cell activation and recruitment [2]. Pro-inflammatory substances are released by neutrophils, and similar activation signals are released by mast cells and other associated cells, which improve vascular penetration and stimulate monocyte migration to the implantation site. There, they undergo differentiation into macrophages, which proliferate endlessly in order to eliminate the biomaterial and then carry out repairs [119]. When implanted biomaterials generate a localized, chronic inflammatory reaction, known as a “foreign body reaction,” macrophages phagocytose the biomaterials and circumvolute them to establish a barrier [120]. Once populated at the lesion site, the macrophages cover and adhere to the implant. Integrins, a family of transmembrane proteins that attach to other proteins and specifically adhere to the proteins on the surface of the implant, perform an essential part in the adherence and covering of the implant. In particular, αMβ2 integrin plays a significant role in the adhesion process due to its ability to bind preferentially to the serum proteins of the implant surface, such as fibronectin and fibrinogen [121]. The cytoskeletal organization of macrophages undergoes a significant shift after attachment. The unified macrophages flatten on the implant surface, engulf and phagocytose it, and expand podosomes, structures that particularly hydrolyze proteins and modify the extracellular matrix. The chemoattractive factors generated by activated and bound macrophages continue to draw in additional macrophages even after the initial implant damage has been healed [122]. The layer of macrophages surrounding the implant created a clear and isolated space. Due to the size of the implant, macrophages cannot phagocytize the whole implant. In order to break down and phagocytize the implant fragments, they consequently release chemicals into this region. When macrophages are unable to phagocytose, they produce degrading enzymes and ROS [123,124].The macrophage attack will pose a major threat to the stability of the implant. Otherwise, stable biomaterials are vulnerable to surface deterioration and fracture due to the damaging inflammatory environment that macrophages create. This can lead to biomaterial resolution or the penetration of toxic substances into the tissue from the lower layer [125,126]. If macrophages successfully degrade and phagocytize the implant during the acute phase of FBR, the response ceases, and the tissue returns to its normal state. As FBR advances into the chronic stage, the macrophage phenotype shifts from the pro-inflammatory type M1 to the anti-inflammatory and tissue generation type M2 [108,127,128]. M2 macrophages also play a key role in FBR-induced fibrosis by drawing fibroblasts to the surface of the implant. As well as reduce inflammation by producing cytokines like IL-10 [129]. Additionally, macrophages aggregate and integrate foreign body giant cells (FBGCs), one of the hallmarks of FBR, to control the subsequent stages of wound healing. The FBR is tightly associated with macrophages, which secrete cytokines and balance various cell types to influence both acute and chronic inflammation [130].

### 3.2. Classification, Manufacture, and Application

#### 3.2.1. Magnesium-Based Biomaterials

Magnesium is a crucial component for human health and longevity due to its involvement in multiple physiological processes, covering cell signaling, ion channel function, biomolecule stability, and cellular metabolism [131,132]. In addition, magnesium exerts an osteo-immunomodulatory impact by stopping conversion to the M1 subtype and encouraging macrophage convergence to the M2 subtype [133,134]. Conversely, a rise in the M2 type has a bone-regenerative impact, while a decrease in the M1 type has an anti-inflammatory effect [135,136]. Therefore, magnesium-based biomaterials are created based on these properties. Its excellent mechanical, biodegradable, biocompatible, and physical qualities make it popular in bone and cardiovascular applications [137]. Electrospun membranes incorporating magnesium oxide (MgO) were manufactured by researchers adopting acetic acid-mediated electrospinning. The results showed the membranes altered macrophage phenotype transition from M1 to M2, raised the amount of TGF-β1, and decreased the expression of pro-inflammatory molecules like IL-1β, IL-6, IL-1α, and TNF-α to reduce inflammatory reactions [138]. Meanwhile, the magnesium ion-chelated nanofibrous membranes (PCL/gelatin/MgAC nanofiber membranes) were designed using a different investigation. Acetic acid was added to polycaprolactone (PCL)/gelatin solutions to convert magnesium oxide (MgO) nanoparticles to magnesium acetate (MgAC) in the process of creating electrospun solutions. It is believed that the anti-inflammatory action can be accomplished via polarizing M2 macrophages and reducing the levels of pro-inflammatory molecules such as TNF-α and IL-1β [139]. Additionally, Jeong et al. created a novel stent known as SRL/MH/PLGA by combining sirolimus (SRL), magnesium hydroxide (Mg (OH)₂, MH), and poly (lactic-co-glycolic acid) (PLGA) with drug-eluting stents (DESs). They employed a solvent casting technique to produce PLGA and PLGA/MH films. The results exhibited a positive anti-inflammatory effect on disease, mainly by lowering the levels of inflammatory cytokines, particularly IL-6 [140]. A material known as magnesium-doped bioactive glass (Mg-BG) was produced by means of incorporating magnesium into BG through the sol–gel technique. The findings showed that Mg-BG has an anti-inflammatory effect by lowering the expression of inflammatory factors, including IL-4, IL-6, IL-8, and TNF-α [141]. Yang et al. developed a novel drug delivery system, namely the magnesium (Mg)/poly (L-lactic acid) (PLLA) composite microsphere, utilizing a water-in-oil-in-water (w/o/w) double-emulsion solvent extraction/evaporation process and employing bovine serum albumin (BSA) as the model protein medication. The results evidenced that this can alleviate inflammation specifically by reducing IL-1β and TNF-α levels and inhibiting the number of inflammatory cells, particularly macrophages [142].

Currently, rat models have demonstrated that magnesium-based biomaterials have a positive impact on the immune system [143]. Following the implantation of a coronary stent, Peng et al. found that the Mg–Zn–Ag system has an anti-inflammatory activity that reduces the requirement for anti-inflammatory drugs [144]. According to another study, magnesium from the Mg–Nd–Zn–Zr alloy can also influence vascular smooth muscle cell (VSMC) activities by controlling macrophage immunoregulation [145]. Moreover, when magnesium-based biomaterials are transplanted into the human body, the amount of FBGCs is comparatively reduced as opposed to polymer-based materials [146,147]. Current research has shown that magnesium-based biomaterials primarily reduce inflammation by altering macrophage-associated cell activity, which promotes macrophage migration from M1 to M2, and downregulating NF-κB signaling can also lessen local inflammatory responses [148,149]. However, a more thorough and targeted molecular study is still required to understand how magnesium-based biomaterials reduce inflammation, particularly in macrophages. Details on the application of magnesium-based biomaterials are given in Table 4.

#### 3.2.2. Keratin-Based Biomaterials

Based on the amount of sulfur, keratin, which is found in hair, wool, nails, and other structures, can be categorized as either soft or hard. Hard keratin makes up the majority of the outer layers of skin, hair, and nails [150]. Because of its excellent anti-biomaterial, biodegradable, antibacterial, antioxidant, biocompatible, and multi-responsive properties, keratin has shown unexpected promise as a biomaterial [151,152]. Consequently, keratin-based biomaterials, such as hydrogels, coatings, and scaffolds, have demonstrated significant promise in tissue engineering and regenerative medicine and are extensively employed in the medical profession (bone, skin, nerve regeneration, etc.) [153,154,155,156,157,158,159]. Keratin-based biomaterials have been shown to promote the polarization of alternatively activated macrophages using in vitro models of monocyte cell lines [153]. Shen et al. reconstituted the lyophilized material with PBS to create a hydrogel. The lyophilized materials were created by removing the hair fibers, neutralizing, centrifuging, and filtering the extracts from human hair, and then purifying, condensing, and lyophilizing them using a freeze-dry method. The inflammatory response in the human body did not considerably worsen after the hydrogel was implanted. Macrophage mannose receptor (MMR)marker expression indicated that M2 macrophages were the most common kind of macrophage [160].

Another study that used human primary macrophages in contact with immobilized keratin biomaterials found that the macrophage phenotype changed and favored an anti-inflammatory phenotype. The finding evidenced that there was a considerable correlation between this alteration and the relative molecular weight of keratin [161]. With regard to the results of this study, motivating macrophages to transition from an M1 pro-inflammatory to an M2 anti-inflammatory phenotype would be a beneficial therapeutic approach for tissue regeneration. Details on the application of keratin-based biomaterials are given in Table 4.

#### 3.2.3. Silk Fibroin-Based Biomaterials

Silk fibroin (SF), which is generally made from silkworm and spider silk, has remarkable mechanical performance, low-grade inflammation, excellent flexibility, biocompatibility, and biodegradability, as well as simple cell adhesion and proliferation, ease of processing, and water penetration [162,163]. These amazing advantages have led to the development of SF-based biomaterials into a wide range of material morphologies, including films [164], hydrogels [165], sponges [166], nanoparticles [167] scaffolds [168], and more. Ruiz-Alcaraz et al. discovered that SF-loaded nanoparticles can significantly reduce inflammation by reducing pro-inflammatory cytokines like TNF-α and IL-6 in macrophages [167]. Another study used a rat model of experimental colitis to further illustrate the anti-inflammatory properties of SF particles [169]. Concurrently, Song et al. observed that SF nanofiber membranes combined with antimicrobial peptides attenuated the expression of inflammatory cytokines, which consequently promoted wound healing [170].

Furthermore, SF is widely used in conjunction with other biomaterials or functionally modified. QK-SF hydrogel, which is composed of SF and mimics vascular endothelial growth factor (KLTWQELYQLKYKGI), has been found by Chen et al. to not only encourage the phenotypic transition of macrophages from M1 to M2 but also to lower inflammation, encourage angiogenesis, and speed wound healing [171]. Also, curcumin nanoparticles (Cur-NPs) were added to sodium alginate and SF(SF/SA) to make hydrogel dressings. Ca^2+^ crosslinked the SF with SA chains to form a three-dimensional (3D) hydrogel network. After that, they made the composite hydrogel dressings by encasing Cur-NPs in the SF/SA hydrogel, which inhibited IL-6 levels for lowering inflammation [172]. The methacrylate-silk fibroin (SilMA)/nano-hydroxyapatite (nHA) composite hydrogel was established by Zhou et al. by first adding methacrylic acid groups to SF and then integrating nHA into SilMA. The investigation concluded that M2 macrophages are stimulated by SilMA/nHA to express relevant genes such as *Arg-1* and *Cd14*. Furthermore, it selectively enhanced M1-to-M2-transitioning macrophage polarization [173]. In addition, researchers devised a novel hydrogel entitled glycidyl methacrylate (GMA)-modified SF (MeSF). Proton nuclear magnetic resonance (1H-NMR) was utilized to evaluate SF after it had been chemically modified with GMA. The results showed that MeSF did not significantly increase inflammation around the implant, according to measurements of inflammatory markers such as TNF-α and IL-1 taken during the experimental test [174]. Babaluei et al. synthesized an enzymatic crosslinking injectable hydrogel (SF/CMC/AG&GO@PDA) by combining the polydopamine-functionalized graphene oxide (GO@PDA) withSF, carboxymethyl cellulose (CMC), and agarose (AG) as the raw ingredients. The study stated that the hydrogel mitigated TNF-α expression to diminish inflammation, in contrast to the control group [175].

The research indicated the anti-inflammatory properties of SF are closely related to the NF-kB signaling pathway [176]. Meanwhile, Kim et al. found that SF can inhibit the expression of cyclooxygenase-2 (COX-2), IL-6, IL-1β, and TNF-α to lessen inflammatory responses and greatly enhance the anti-inflammatory properties of Tat-SOD [177]. Nevertheless, no more studies have been conducted to comprehend the precise anti-inflammatory mechanism of SF, which leads to the complex relationship between inflammation and SF still being unknown. Therefore, further thorough research on the anti-inflammatory mechanisms of SF is still required for greatly using the anti-inflammatory qualities of SF-based biomaterials. Details on the application of SF-based biomaterials are given in Table 4.

#### 3.2.4. Flavonoid-Based Biomaterials

Flavonoids are produced from polyphenolic plants and can be found naturally in fruits, vegetables, flowers, stems, roots, leaves, grains, tea, berries, and olive oil [178]. A few of their many attributes include anti-inflammatory, antibacterial, immune-regulating, anti-cancer, antiviral, and anti-apoptotic properties [179,180]. The structure of flavonoids is thought to be responsible for their potent anti-inflammatory effects. Based on their constituents and molecular structures, various subclasses of flavonoids, such as flavanones, flavones, isoflavones, flavanones, anthocyanins, and chalcones, can be distinguished [178,181]. A variety of mechanisms contribute to the anti-inflammatory effect. Currently, studies have shown that inhibiting pro-inflammatory mediators assists in decreasing inflammation in cellular models or in vitro. Multiple flavonoids have been proven in studies to lower the amount of pro-inflammatory chemokines in RAW macrophages, peripheral blood mononuclear cells, and Jurkat T cells, like TNF-α, IL-6, IL-8, and monocyte chemoattractant protein-1 (MCP-1) [182].

Flavonoids have been shown to activate phase II antioxidant detoxification enzymes, MAPK, Nrf2, and protein kinase C while also suppressing the activating protein-1 (AP-1) transcription factor [182,183,184]. Additionally, several flavonoids reduce the levels of prostaglandins, leukotrienes, and NO by acting as inhibitors of cyclooxygenase, NOS, phospholipase A2, and arachidonic acid. For example, Genistein, Kaempferol [178], Quercetin, and Apigenin [185] can suppress COX-2, while Luteolin, Quercetin, and Apigenin can decrease NO synthase [178]. Moreover, additional pathways that flavonoids can regulate to lower inflammation include those related to NF-kB, MAPK, ERK, PI3K/Akt, and protein kinase [186]. Apart from that, it has been demonstrated that flavonoids reduce neutrophil aggression and regulate neutrophil actin polymerization [179]. They can suppress the formation of arachidonic acid metabolites and chemokines, which can reduce inflammation by restricting complement system activation [187], lower ROS levels [188], and lessen white blood cell edema and exudation [189]. Thus, flavonoids can be employed for treating various kinds of diseases, including rheumatoid arthritis (RA), neurological disorders, IBD, and retinal degeneration, corresponding to their excellent benefits [186]. Although its anti-inflammatory qualities have been shown in plenty of trials, relatively few in vivo studies have been conducted. Consequently, greater in-depth research is a key concern in the usage of flavonoids.

Applying a multilayer molding methodology, Fang et al. set up a new conduit described as fisetin/chitosan/polycaprolactone nerve guide conduits (FIS/CS/PCL NGCs) by incorporating fisetin-loaded chitosan hydrogels into the lumen of polycaprolactone nerve guide conduits. IL-1 and CD 68 expression did not substantially vary between the autograft and nerve guide conduit groups, as indicated in the research, which could hinder an increase of M1 macrophages as well as minimize inflammatory reactions [190]. Establishing GSC via photo-cross-linking gelatin methacrylate (GelMA) and secondary copper-cross-linking sodium alginate (SA). Subsequently, lutein nanoparticles were added to the polymeric carrier PBE, a substance with benzyl borate ester and tertiary amine groups. A double network hydrogel designated GSC/PBE@Lut came into being. The results demonstrated GSC/PBE@Lut could eliminate ROS, encourage macrophage polarization toward M2, and prevent macrophage polarization toward M1. Added to that, it diminished the expression of pro-inflammatory factors like TNF-α and IL-6 and raised the expression of anti-inflammatory factors like IL-10. All of them contributed to an overall reduction in inflammation [191]. In this context, Wei et al. invented quercetin-loaded liposomes modified with galactosylated chitosan (GC-Que-Lipo). Encapsulating quercetin in liposomes and subsequently attaching them to GC, which was manufactured by electrostatically connecting chitosan and lactobonic acid. In accordance with the study, invasive and migratory macrophage counts dropped, the anti-inflammatory factor IL-10 levels rose, while the pro-inflammatory factor TNF-α dropped. These consequences could boost M2 macrophage polarization along with alleviating inflammation [192]. Similarly, another study developed a material called quercitrin-nanocoating by grafting quercitrin onto titanium (Ti) surfaces. In both basal and inflammatory circumstances, this compound effectively decreased functional product prostaglandin E2 (PGE2) and inhibited COX2 expression. Quercitrin also possesses antioxidant properties. These would lessen inflammation [193]. Additionally, using an immersion-precipitation phase transition technique, Wang et al. created a membrane known as a silymarin (SM)-modified poly-sulfone (PSF) hemodialysis membrane. The study demonstrated that SM/PSF membranes facilitated suppressing the transition of M1 macrophage phenotypes. As well as playing an important role in mediating inflammatory reactions to inhibit nitric oxide generation and *iNOS* gene expression [194]. Details on the application of biomaterials based on flavonoids are given in Table 4.

**Table 4:** Application of biomaterials targeting macrophages in diseases.

	Disease	Designed Materials	Actions	Reference
Magnesium-based biomaterials	Wound healing	Electrospun membranes incorporating MgO	(1) Altering macrophage phenotype transition from M1 to M2 (2) Raising the amount of TGF-β1 (3) Decreasing the expression of pro-inflammatory molecules like IL-1β, IL-6, IL-1α, and TNF-α	[138]
	Diabetic wound healing	PCL/gelatin/MgAC nanofiber membranes	(1) Promoting polarizing M2 macrophages (2) Reducing the levels of pro-inflammatory molecules such as TNF-α and IL-1β	[139]
	Cardiovascular diseases	SRL/MH/PLGA	(1) Lowering the levels of inflammatory cytokines, particularly IL-6	[140]
	Inflamed pulps	Mg-BG	(1) Suppressing the expression of inflammatory factors, including IL-4, IL-6, IL-8, and TNF-α	[141]
	Drug delivery	Mg/PLLA composite microsphere	(1) Reducing IL-1β and TNF-α levels (2) Inhibiting the number of inflammatory cells, particularly macrophages	[142]
Keratin-based biomaterials	Cardiac dysfunction after myocardial infarction	Keratin hydrogel	(1) Failing to worsen the inflammatory response (2) Increasing MMR marker expression of M2 macrophages	[160]
Silk fibroin-based biomaterials	Bacterial-infected wound healing	SF/SA hydrogel	(1) Inhibiting IL-6 levels	[172]
	Bone regeneration	SilMA/nHA hydrogel	(1) Promoting macrophage polarization from M1 to M2 (2) Stimulating M2vmacrophages to express relevant genes such as *Arg-1* and *Cd14*	[173]
	Alveolar bone defect	MeSF	(1) Not considerably raising inflammation according to the expression of inflammatory markers such as TNF-α and IL-1	[174]
	Full-thickness burn healing	SF/CMC/AG&GO@PDA hydrogel	(1) Mitigating TNF-α expression	[175]
Flavonoid-based Biomaterials	Peripheral nerve injuries	FIS/CS/PCL NGCs	(1) Not substantially changing IL-1 and CD 68 expression (2) Hindering an increase of M1 macrophages as well as minimizing inflammatory reactions	[190]
	Wound healing	GSC/PBE@Lut	(1) Eliminating ROS (2) Encouraging macrophage polarization toward M2, and preventing macrophage polarization toward M1 (3) Diminishing the expression of pro-inflammatory factors like TNF-α and IL-6 and raising the expression of anti-inflammatory factors like IL-10	[191]
	Acute liver injuries	GC-Que-Lipo	(1) Dropping the number of invasive and migratory macrophages (2) Rising anti-inflammatory factor IL-10 levels while decreasing the pro-inflammatory factor TNF-α (3) Boosting M2 macrophage polarization	[192]
	Dental implants	Quercitrin-nanocoating	(1) Decreasing functional product PGE2 and inhibiting COX2 expression	[193]
	Blood purification	PSF/SM membranes	(1) Suppressing the transition of M1 macrophage phenotypes (2) Playing a crucial role in mediating inflammatory reactions to inhibit nitric oxide generation and *iNOS* gene expression	[194]

In conclusion, biomaterials based on magnesium, keratin, silk fibroin, and flavonoids specifically promote macrophage phenotype transformation to M2 while suppressing transformation to M1, thereby reducing inflammation. Additionally, these biomaterials can mitigate inflammation by inhibiting the expression of pro-inflammatory factors such as IL-1β, IL-6, IL-12, TNF-α, and IFN-γ and contributing to the expression of anti-inflammatory ones such as TGF-β, IL-10, and IL-13.

## 4. Summary and Future

Given the growing application of biomaterials in inflammatory diseases as a feasible and efficient therapeutic strategy and the closely associated connection between ROS, macrophages, and inflammation, we discuss biomaterials that specifically target macrophages and ROS.

In this review, we covered how biomaterials regulate macrophages and scavenge ROS to reduce inflammation. Scavenging ROS biomaterials can be useful by directly contacting ROS, suppressing ROS production, and accelerating ROS clearance. Additionally, anti-inflammatory effects are produced by biomaterials regulating macrophages, mainly by changing their polarization. Four categories of biomaterials—magnesium-based, keratin-based, silk fibroin-based, and flavonoid-based—that control macrophages are the subject of this investigation. These four biomaterials have the ability to decrease inflammation by promoting macrophage polarization to M2 and inhibiting polarization to M1. Furthermore, inflammation can be reduced by boosting the expression of anti-inflammatory factors like TGF-β, IL-10, and IL-13 and suppressing the expression of pro-inflammatory factors like IL-1β, IL-6, IL-12, TNF-α, and IFN-γ. In addition, our study demonstrated that there are not many comprehensive and precise studies on the anti-inflammatory effects of biomaterials based on magnesium and silk fibroin. In order to employ biomaterials to reduce inflammation in the future, a more thorough investigation is therefore required.

Taking everything into account, the present developments in biomaterials that control macrophages and scavenge ROS for the treatment of inflammatory illnesses have progressed swiftly, presenting a multitude of possible applications for anti-inflammatory biomaterials. The future of anti-inflammatory biomaterials is still radiant despite the fact that many questions remain. We anticipate that this review will provide useful information for the biomedical field and relevant research.
